# Factors that influence women’s enrolment and ongoing participation in a partially decentralised randomised controlled dermatology trial: a qualitative interview study with participants in the SAFA (Spironolactone for Adult Female Acne) trial

**DOI:** 10.1186/s13063-023-07630-4

**Published:** 2023-10-12

**Authors:** Cherish Boxall, Susanne Renz, Zina Eminton, Jacqueline Nuttall, Alan Saji, Charlotte Cluff, Christopher Wilcox, Ingrid Muller, Alison M. Layton, Irene Soulsby, Miriam Santer

**Affiliations:** 1 Southampton Clinical Trials Unit, University of Southampton, Southampton, SO16 6YD UK; 2 Faculty of Medicine, University of Southampton, Southampton, SO16 6YD UK; 3 Primary Care Research Centre, University of Southampton, Southampton, SO16 6YD UK; 4 Skin Research Centre, University of York, York, UK

**Keywords:** Decentralised, Hybrid, Experience, Trial, Dermatology, Recruitment, Retention, Qualitative

## Abstract

**Background:**

The use of decentralised clinical trials (which bring trials to patients through remote processes and technology versus central on-site visits) has been thought to be a potential solution to common recruitment and retention barriers. However, there is a lack of evidence to understand the experiences, needs and preferences of the public to inform trial methodologies that appeal to different populations. We report participant experiences of SAFA, a partially decentralised randomised clinical trial, to inform the methodology used in future dermatology trials that aim to appeal to women aged 18 and over.

**Methods:**

Participants of the SAFA (Spironolactone for Adult Female Acne) trial were invited to take part in a qualitative semi-structured interview to explore their experience and perspectives of taking part in the trial. Questions focused on their experience of using decentralised methods to access and enrol in the trial (e.g. social media advertising), in addition to the decentralised trial visit and data collection methods used throughout. Interviews were conducted remotely, recorded, and transcribed. Data were analysed using reflexive thematic analysis.

**Results:**

Twelve SAFA participants (all women, age range 22–36 years) were interviewed. Initially, participants were influenced to enrol by trusted online information, the feeling of validation the trial provided, and the convenience and flexibility offered by the decentralised methods and research staff made participants feel valued and enabled them to engage in the trial with minimal interference to existing commitments. SAFA participants were generally accepting of trial demands, such as the text-heavy paperwork and on-site visits for blood collection and highlighted several areas relevant for trial conduct going forwards including where decentralised methods may (and may not) be accepted and how trial accessibility and understanding could be improved.

**Conclusions:**

The study has shown that decentralised methods used by responsive and approachable staff were widely accepted in the SAFA trial. Interviewees found the methods adopted in the SAFA trial helped the trial to fit with their needs and promoted a sense of feeling valued that encouraged ongoing trial engagement. Decentralised methods should be considered favourably when designing a dermatology trial as they can potentially enhance both recruitment and retention.

**Trial registration number:**

ISRCTN 12892056. Registered on October 15, 2018.

**Supplementary Information:**

The online version contains supplementary material available at 10.1186/s13063-023-07630-4.

## Background

People’s willingness to volunteer their time to participate in clinical trials is crucial to the development of evidence that advances clinical treatment and healthcare practice. Recruitment and retention of participants have continued to be two of the largest challenges to date, with over half of the trials failing to meet recruitment targets, resulting in insufficient data, trial delays, increased costs and ethical concerns [1]. 

Known barriers to participants enrolling on trials include fear of perceived risk, transport, time, distrust, and aversion to randomisation [2]. Moreover, difficulties in scheduling and attending in-person visits are significant reasons for low recruitment and may be a barrier to enrolment for those in full-time employment or with caring responsibilities [3].

Cochrane systematic reviews have shown there is a need to generate evidence-based solutions to these common barriers and to encourage participants to enrol and continue participation in research [4, 5]. The need to generate evidence is further supported by two recent James Lind Alliance prioritisation processes, Prioritising Recruitment in Randomised Trials Study (PRioRiTy I, 2018; [6]) and Prioritising Retention in Randomised Trials Study (PRioRiTy II, 2019; [7]). The PRioRiTY I and II projects involved collaboration with public contributors and professionals to identify and prioritise unanswered questions relating to trial recruitment and retention, respectively.

The use of decentralised trials (which bring trials to patients through remote processes and technology versus requiring them to visit a central research site [8]) has the potential to be a cost-effective solution to common recruitment and retention barriers. Decentralisation can be applied fully or partially, the latter often is referred to as hybrid, which means some conventional methods (e.g. in-person visits to a research site) are used alongside decentralised approaches (e.g. electronic patient-reported outcomes).

COVID-19 has been the catalyst for a 93% increase in the use of digital technology to conduct decentralised trials as an alternative to in-person visits between 2020 and 2021 which is continuing to grow [9, 10]. However, although there may be a great benefit to enabling participants to take part in trials outside of the research site (e.g. from home) by overcoming common recruitment and retention barriers, there is a lack of evidence to understand the experiences, needs and preferences of the public to inform best practice [3].

Many dermatology trials are considered non-life threatening and rarely require complex examinations, leading some to believe that decentralised trials may be an attractive option for this type of research [11]. However, there is a lack of qualitative enquiry in this patient population to explore whether decentralised trials are acceptable to participants.

This qualitative study aimed to explore the motivators and barriers to initial enrolment and ongoing participation in the partially decentralised dermatology trial called SAFA (Spironolactone for Adult Female Acne) and contribute to the following James Lind Alliance PRioRiTy questions on recruitment and retention, respectively [12, 13]:

- What are the key motivators influencing public decision to take part in an RCT?
- What motivates a participant to complete a clinical trial?

## Method

### Setting

**Fig. 1:**
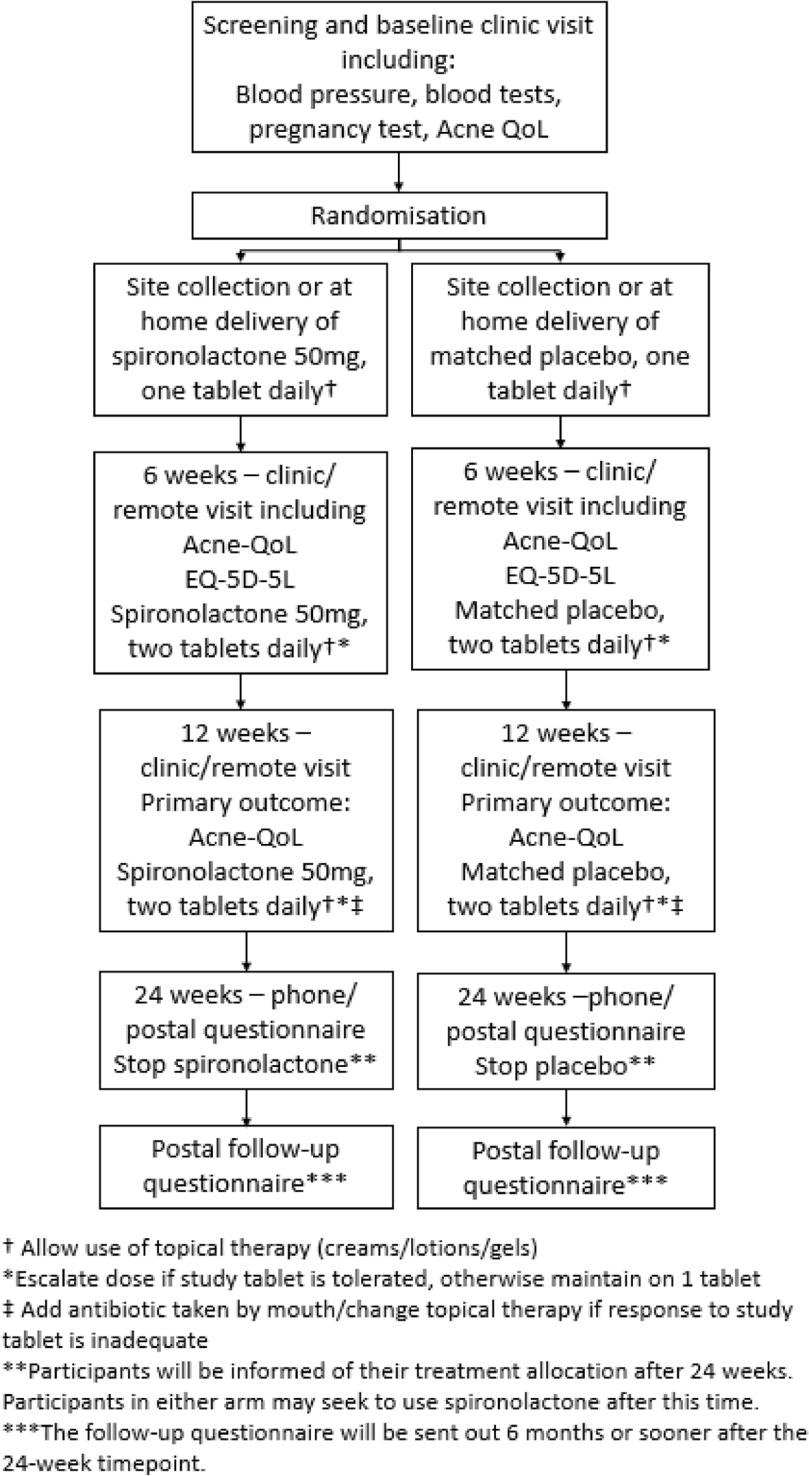
SAFA trial schema

This was a qualitative sub-study conducted alongside a UK primary and secondary care phase III, double-blind, placebo-controlled trial of Spironolactone for persistent (at least 6 months) facial acne vulgaris for women aged 18 and over (Spironolactone for Adult Female Acne (SAFA) trial, Fig. 1) [14]. A total of 410 SAFA participants were randomly assigned (1:1) to either 50 mg/day spironolactone, increasing to 100 mg/day from weeks 6 to 24 or matched placebo. Spironolactone was provided in tablet form, and participants were able to continue with topical treatments. Published results from the trial concluded that spironolactone significantly improved outcomes, with a greater difference at week 24 than at week 12 [15].

The SAFA trial recruited participants from 05 Jun 2019 to 31 Aug 2022, with an enforced pause to recruitment from 23 Mar 2020 to 11 Jun 2020 due to the COVID-19 pandemic. Participants were recruited through primary care (search and mail-out or opportunistic recruitment), secondary care (opportunistic recruitment) and community and social media advertising.

The SAFA trial was designed as a pragmatic trial with minimal burden (e.g. in-person visits, number of spironolactone retrievals from pharmacy) for participants, and required three in-person visits (baseline, 6 weeks and 12 weeks) with postal questionnaires at 24 weeks and up to 52 weeks. Due to the COVID-19 pandemic, the trial further decentralised to reduce in-person contact, specifically: all visits except the baseline visit could be replaced with telephone calls or video calls, acne assessment photos could be taken and sent to staff by participants, and trial medication that originally required on-site collection by participants was delivered to their home where necessary. Baseline visits required blood and pregnancy tests, so these remained in-person visits at research sites. These changes were sustained beyond government-enforced lockdown for the duration of the trial.

This qualitative sub-study aimed to explore participants’ experiences of taking part in the SAFA trial, in particular their experience of recruitment to the trial, the experience of appointments during the trial and/or telephone/video appointments and any difficulties participating in the trial to inform dermatology trial methodology targeted towards women aged 18 and over. Standards for reporting qualitative research (SRQR) guidelines have been followed (Supplementary file 1).

### Sampling strategy

Participants enrolled in the SAFA trial were invited to take part in an optional qualitative interview with no monetary incentive. Participants were invited to take part opportunistically (based on their willingness and ability) by research staff at sites or by unblinding letters at 24 weeks. The total number of invited participants by research sites is unknown because they were not requested to keep a log, and 174 were invited through an unblinding letter sent 28 weeks after their baseline visit. Trial participants who were interested in being interviewed were asked to email the SAFA qualitative study mailbox accessed by the researchers conducting interviews (CB, AS, CC), who then responded to them via email to provide the Qualitative Research Information Sheet and Qualitative Interview Consent Form. There was no decline slip, therefore the reasons for not taking part are unknown. Once a hand or electronically signed consent was received, the interview time was confirmed and subsequently conducted by telephone or video call (participant preference). Participants were asked if they had questions during the recruitment process and again immediately before the interview.

The qualitative sub-study was not funded as part of the trial and resources and ethical approval for this only later became available. Trial recruitment ran from June 2019 to August 2021, whilst invitations for qualitative interviews started in August 2021.

### Interviews

To understand and interpret the subjective experiences and perspectives of the individuals being interviewed, and acknowledge the researcher’s role in shaping the meaning of the findings, the research questions were addressed with a constructivist approach [16]. Semi-structured interviews followed a guide developed by the study team, with input from a patient representative to capture the experience of taking part in the SAFA trial. Specifically, questions focused on their views and experience of the social media advert, the video/telephone appointments, and data collection methods.

Three members of the research team (two students and one research fellow), independent from the SAFA trial, conducted the interviews (CB, AS, CC) from October 2021 to February 2022. All identifiable data were anonymised, and quotes were labelled with pseudonyms at the write-up. The duration of the interviews was on average 28 min (range 19–39). All interviews were transcribed verbatim, error-checked and data were handled using NVivo10.

## Analysis

Reflexive thematic analysis [17] involves identifying and analysing patterns across the interviews and was applied to analyse the data because of its alignment with the principles of constructivism, in particular the shared emphasis of exploring participants’ viewpoints whilst acknowledging the researchers’ role in interpreting the data. Reflexive thematic analysis was also flexible to the opportunistic sample and facilitated the discovery of rich and complex understandings that could transfer to similar contexts. Once the transcripts had been read through for familiarisation, the data were coded by CB using NVivo10 software and initial themes were then generated from coding commonalities and refined through continued reading, analysis and discussion with MS and IM. Themes were developed by recognising concepts directly communicated by participants, with subsequent consideration of deeper connections and patterns through researchers’ interpretations during sense making of the findings. A lack of disconfirming data suggests there were similar experiences shared by the target group, and information power was judged to have been achieved in understanding participants’ views and experiences of the trial [18].

## Ethics

Ethical approval for the trial was granted by Wales Research Ethics Committee 3 in January 2019 (reference number: 18/WA/0420). An amendment approving the qualitative sub-study was granted in August 2021.

## Results

Twelve SAFA trial participants completed an interview (Table 1). Three main themes were identified: (1) the influence of trust when deciding whether to enrol, (2) the feeling of validation from start to end and (3) offsetting participant burden with the understanding of trial needs.

**Table 1:** Characteristics of interview participants

**Total number**	12
**Mean age in years (range)**	29 (22–36)
**Ethnicity**	
White	10
Mixed or multiple ethnic groups	1
Prefer not to say	1
**Occupation**	
Paid employment	10
Self-employed	1
Unemployed	1
**Type of trial medication**	
Spironolactone	6
Placebo	3
Unsure (not yet unblinded)	3
**Route of recruitment**	
Facebook/Instagram/Twitter	5
Primary care	2
Secondary care	1
Poster	2
Online chatroom	1
Searched online for trials	1

### Theme 1: the influence of trust when deciding to enrol

This first theme describes participants’ motivators for enrolling on the trial and factors that they described as important in this. Many participants had tried and tested new treatments for acne off the shelf or through their doctor, but due to a lack of effective results continued to read about possible treatment options on websites and online acne communities.

Many participants belonged to Facebook groups, Reddit forums and reported using the internet to contribute and read about acne treatments, which led some to be aware that Spironolactone is used as a treatment in America.

I found out about it [Spironolactone] on Reddit..it’s sort of like a forum/social media site. The way it works is there’s different communities on there, where people can put up posts and share things. There’s one dedicated to skincare and I’m part of a worldwide skincare one. Katy, age 27

Due to the trusted information received from this source, several participants were already aware of the use of Spironolactone for acne and unsuccessfully sought it from their GP. It is possible that when they became aware of the SAFA trial, they saw an access point to a new but familiar treatment. This familiarity may have contributed to their low level of safety concern.

I’d been reading about spironolactone and hearing about it, because it’s used widely in America. I’d actually tried to acquire some off licence, through my GP, years ago. I actually thought it was a very good thing, because I’d already known about it - then the success they’ve had in America, where it is used widely. Natalie, age 27

Had the drug been an unknown one, I would have not been so keen to take part. Helen, age 30

Participants felt that advertisements for the trial were produced to a high standard of quality and not something easily produced by the layperson, which in combination with the affiliated University and NHS logos, were a strong sign of legitimacy.

It was at the University of [University Name], or [Hospital Name] Hospital, so yes, went and just applied. Sam, age 25

Participants that enrolled through social media felt that it provided a safe, low-effort and direct access point to research from home and a seamless transition into the trial.

I was worried there might be loads of hoops to jump through and it would end up dragging on for so long that I’d give up! But actually it was really quick and easy to get involved. Laura, age 36

One concern reported by participants was the self-taken digital photo used to assess the severity of their facial acne and eligibility for the trial. Many worried the photos would not provide an accurate representation of their skin due to the lighting or picture clarity, with concerns that the severity of the acne might not be recognised.

I had to take my own photos of my skin and send them in for the doctor to review, and then you’ve got, obviously, the deep concerns for my end, as did I get the right photos? Are they clear enough?..it’s very different sending in a photo than someone actually physically looking at your skin face-to-face. Linda, age 32

Concerns of inaccurate representation raise issues around participants’ trust in the digital assessment process and subsequent validation of the severity of their acne.

### Theme 2: the feeling of validation

This second theme describes how participants felt a sense of validation from the opportunity to take part in the SAFA trial. Many participants reported that previous treatment had been ineffective and felt that the burden of acne was not considered as serious by their GP. Access to the SAFA trial appeared to counter these feelings and made patients believe their acne was being ‘taken seriously’.

Some women, before becoming aware of the SAFA trial, had approached their GPs and, unsuccessfully, requested Spironolactone as a treatment option. Once enrolled participants said they felt grateful that they could try a new treatment and receive access to specialist support that they otherwise may not have had.

My GP had never put me forward to go to the hospital. She’d always just said try different things. Seeing a nurse who is like a dermatologist specialist, and like the doctor, was really useful. Jasmine, age 22

Participants described how, from initial contact, site research staff delivering the trial had a significant impact on their experience. Participants discussed how they felt valued when they received a quick response to their online expression of interest, which included being talked through the trial remotely or in person by a member of the research team and the opportunity to ask questions.

I found that the team at the Hospital were really friendly and welcoming.. I was also kept informed by the team at the hospital about next steps, and that was explained really well to me. I felt like they were really welcoming for me to ask questions as well. I had a really positive experience. Katy, age 27

The importance of making time to talk to participants was highlighted when one woman shared her experience of feeling bombarded with medical jargon, questions, and a lot of information during her in-person visits. She justified the experience by believing it was the nature of the job and the strain COVID had put on the staff.

The lady who ran those [in-person visits], she sometimes was practically running in front of me from room to room and it was just all very like [makes sound], it’s quite hectic.. I just needed a bit of time to go through it a bit slower with me and help me understand what they were saying. Emily, age 26

Participants appreciated being provided with the choice of how to complete their follow-up trial visit (e.g. in-person or remotely). Providing the option allowed many to overcome work, care and travel barriers in a time-efficient manner and because the trial and research teams worked around them, it made them feel as though their input was valued.

..I was getting all the kids in the car ready for the school run and she was like, ‘Are you sure you can talk now?.. She was always happy to call back or call around what I was doing. So that was really good; very flexible. Kat, age 30

### Theme 3: offsetting participant burden with the understanding of trial needs

Study participants reported an’ overwhelmingly positive experience and trust in the research staff, which often led them to overlook minor inconveniences. However, when asked directly about certain aspects of the trial, and more broadly, what could be done to improve their experience there were several suggestions. This third theme explores how trials may be improved from participants' experiences.

Some participants described the ‘massive booklet about the study’ (the participant information sheet) they received as part of the introduction to the trial as ‘intense’ and ‘complicated’ which caused them to delay reading it. However, all appeared to understand the purpose of the information sheet. One participant with dyslexia showed consideration for those who may have additional learning needs or were slightly less confident when accessing and understanding the information in the written information sheets.

The information sheet..was quite wordy! For people that maybe have additional learning needs, they may have benefitted from maybe bigger text, or different colour text on different coloured background, but that's just me putting a dyslexic mind to it. Emily, age 26

It was the combination of the written information and clear explanation with research staff that enabled participants to understand different components of the trial, including randomisation, trial tasks and the possibility of receiving a placebo.

When asked about their feelings regarding the possibility of being randomised to placebo, most hoped they were on the active drug but also that they ‘knew exactly what they were signing up for’. Despite the potential disappointment of receiving the placebo, participants felt it was important that they were contributing to finding better treatments for women with acne.

I was on the placebo then I still would have taken part in something that ultimately will help people with acne, so yes, I wasn’t upset about being on the placebo either because it’s gone a long way, I hope, to help the medication being more available for other women with acne. Emily, age 26

One participant, who believed she was receiving the placebo because the drug she received did not match descriptions of Spironolactone on the internet, committed to the trial for 6 weeks before leaving because she felt it was not a good use of personal time. This individual was subsequently prescribed Spironolactone and found the treatment effective by 3 months.

I’ve been dealing with this for the past two to three years now; you get to a point where unfortunately I just didn’t feel that I had that time to spare. As much as I’d like to help medical research, it had gone on long enough for me and I needed a solution by that time. Liz, age 36

When asked about the questionnaires, the belief in the research team led many to feel that the volume of data being collected and the questions themselves, although an inconvenience, must be needed and generally accepted as being ‘part and parcel’ of the study.

I mean one thing that was a bit of a pain with it, but, again, it’s one of those things where like it’s part of the process and it has to be done, was that one of the questions felt quite redundant and repetitive. Natalie, age 27

On reflection, participants felt they could have provided more accurate questionnaire answers to reflect any change if they were able to keep their completed questionnaires.

It was quite difficult when I didn’t have my previous set of answers to actually even remember where, on this arbitrary scale, I had placed myself. I was giving a number, but I was like, ‘Is that higher or lower than the number I gave last time?’ because I want it to be lower or I want it to be higher. Shok, age 24

Last, although accepted as a consequence of participating in the trial, on-site visits to provide blood samples and collect research drugs from the pharmacy brought up frustrations around hospital parking and the long period such appointments took.

to have my bloods taken at phlebotomy took 40 minutes extra out of the appointment time. Linda, age 32

The parking at the hospital’s not easy! It’s quite expensive. It was a little bit challenging getting an appointment around work. Kat, age 30

## Discussion

This paper described the experiences of participants taking part in a partially decentralised dermatology trial [14]. The key motivators for initial enrolment and ongoing engagement in the trial were a trusted and efficient access point provided by social media advertising and the sense of value created by flexible trial visit times and contact methods that suited the participant’s needs.

For many interview participants, their first interaction with the SAFA trial was through the social media advert. Findings from this study show that trusted affiliations and the perceived quality of the social media advert increased initial trust, which is a particularly important motivator that should be focused on during social media advertising campaigns [19]. A paper on the SAFA trial social media campaign is being published separately [20].

In a broader context, the themes of trust and validation align with a recent overview of systematic reviews focused on psychosocial barriers and facilitators [2]. Findings revealed several key factors that play a significant role including trust in the research process, the convenience and minimal burden associated with participating in the research, and the potential for personal benefits such as gaining access to innovative treatments or acquiring valuable knowledge. Notably, the prospect of personal benefits could have been a heightened motivator for these participants due to the compounded challenges of managing acne and the lack of truly effective treatment options.

Commonly reported barriers to trial participation include fear, perceived risk and practical difficulties [2] which could have been overcome by the participants existing familiarisation with the trial medication and the pragmatic and convenient trial design. Specifically, the decentralised methods such as the delivery of trial medication and remote (phone or video call) visits reduced travel and time commitments [11] and could have contributed to the successful recruitment of participants of a working age who might have been unable to take time off work or negotiate other commitments [15].

Building upon the significance of trust and validation it’s important to discuss the comprehension of the different components of the trial and its role in participation and engagement. A recent meta-analysis revealed that the proportion of participants in clinical trials who understood different components encountered during informed consent (e.g. placebo, randomisation, study purpose) ranged from 52 to 76% [21]. Despite many feeling familiar with the trial medication, SAFA participants were grateful to the staff who took the time to explain what was involved in taking part in the trial, which consequently could have proactively targeted behaviours linked to poor retention such as knowledge and social influence [22]. Staff communication was also seen to offset the intimidating impression given by the written participant information sheet, a finding that highlights how information is communicated could influence a participant's accessibility and understanding of the trial, a consideration in contention with the text-heavy regulatory requirements, and a consistent balancing act within research communities [23].

Also, interview data from this study suggest that participants believed they may be able to provide more accurate data in follow-up questionnaires if they had access to previously completed versions. The impact on questionnaire validity should be carefully considered alongside existing guidelines on optimal questionnaire design and administration to enhance completeness in a clinical trial [24].

A primary concern raised by participants was around the photographic acne assessment. A recent meta-analysis suggested that ‘teledermatology’ diagnoses are less reliable than those made in person [25].

Future trials may consider the use of image validation software that can provide instant feedback to participants, and participants should be assured that where there is doubt, they will be invited to attend a face-to-face appointment.

### Strengths and limitations

The strengths of this study include its novelty and potential application and impact in practice. To our knowledge, this is one of the first trials exploring the impact of decentralised methods on participant enrolment and ongoing engagement in a dermatology trial.

Some limitations of this study should be considered. We captured the experiences of SAFA participants who volunteered, without incentive, to take part in this qualitative sub-study and for this reason, there may be some inherent selection bias in the attitudes of those who took part. We did not interview eligible participants who chose not to enrol, who may have had different views from those who chose to participate in the qualitative interview. Non-recruited potential participants may have been able to provide insight into deterrents of participation to understand what may be adapted to make the trial more attractive to take part in. In addition, some interviews were carried out up to 8 months after a participant had completed the trial, which made some details of the trial hard to recall for some participants (e.g. details of the questionnaire). A larger sample size may have raised previously uncovered topics of interest. For this reason, future evaluations should be included, where possible, from the trial start with participants and non-participants. Participants should also be allowed the opportunity to share why they have declined the interview, which could inform recruitment strategies.

### Future research

Continued efforts should be made to understand what and how different trial conduct methods can improve accessibility and understanding to enhance trust and influence enrolment and ongoing engagement in trials.

The role of infographics and videos during recruitment to improve viewer understanding is a growing area of research [26], however, the measured impact it has on the participant and subsequent trial recruitment and retention are lacking. Context-specific research is required to understand what the most accepted and effective communication mediums, including content, are. Future trials aimed at this population should aim to compare the impact of communication mediums on participant understanding, enrolment and engagement by embedding a study within a trial, protocols of which can be found in the SWAT repository hosted by The Northern Ireland Hub for Trials Methodology Research [27].

Also, with consideration for ethical issues [28], there may also be merit in exploring the potential to interact with online patient communities to improve understanding, access to trials, and the mobilisation of easy-to-understand evidence-based knowledge.

Some participants in this study expressed frustrations when they did need to attend the hospital site for trial urine and blood tests, which highlights a relevant consideration for trial methodology going forwards. Home-based blood sample collection and drug delivery to home have recently increased in popularity [29] and may serve to reduce the time commitment required by trial participants. To further reduce participant burden and assist with the unprecedented challenges faced by the UK clinical research delivery system [30], further research is needed to understand the cost implications for developing and adopting decentralised methods, and if and how they could be optimised and implemented into practice whilst maintaining public trust and the integrity of the data collected [31].

## Conclusions

If applied appropriately, the use of decentralised methods has the potential to influence the enrolment and ongoing participation of dermatology trial participants and help address well-known recruitment and retention issues amongst research communities [4, 32]. The convenience and flexibility experienced by the participants were brought about by approachable research staff using decentralised methods to run the trial. Such methods should be considered favourably when designing a dermatology trial if careful consideration is given to provide reassurance when decentralised methods (e.g. photos) are used as part of screening and eligibility.

This study was aimed at women aged 18 and over, the key practical findings will therefore be of interest to trialists and healthcare professionals wishing to target this population for future interventions.

### Supplementary Information

**Additional file 1. **

**Additional file 2. **

## Data Availability

The datasets generated and/or analysed during the current study are not publicly available to protect the identity of the participants but are available from the corresponding author on reasonable request. The standards for reporting qualitative research and topic guides used to inform the interviews are available as supplementary material. Please direct all data enquiries to the corresponding author.
