# Supplementary material for: Factors that influence women’s enrolment and ongoing participation in a partially decentralised randomised controlled dermatology trial: a qualitative interview study with participants in the SAFA (Spironolactone for Adult Female Acne) trial
Source: Trials. 2023 Oct 12;24:661. doi: 10.1186/s13063-023-07630-4 (PMC10568833; doi:10.1186/s13063-023-07630-4)
Supplement: Supplementary file 2 — Additional file 2. [file 13063_2023_7630_MOESM2_ESM.zip › SAFA Qualitative Interview Guide v1 25-JUN-2021R2.pdf]

REC Number: 18/WA/0420
IRAS ID: 246637

SAFA study – Qualitative Interview Guide

SAFA: Spironolactone for Adult Female Acne: pragmatic multicentre double-blind randomised superiority trial to investigate the clinical and cost-effectiveness of spironolactone for moderate or severe persistent acne in women

The following topics/questions may be discussed in this study. The qualitative work will remain flexible with respect to participants' agendas but we will cover the broad topics/questions noted. It is common in qualitative work to iteratively develop topics and questions as new ideas emerge from early data collection. Therefore, we may add new topics as the interviews progress and data collection continues. However, the key topics of patient views and experiences on the SAFA study/accessing spironolactone will remain the same.

Written consent will be sought prior to the interview

Introduction:

- Re-introduce self and purpose of interview
  - We are trying to find out, in your own words, as much as possible about how you found taking part in the SAFA study, including negative things or positive things, so we can change it for the future.
  - Have you read the information sheet about the interview study?
  - Do you have any questions before we start?

- Remind the participant
  - the interview will take approximately 30-45 minutes
  - responses will be kept confidential; any direct quotes will be used anonymously and will not identify you
  - You can change your mind about taking part or stop the interview at any time without giving reason. You don't have to answer any question if you don't want to.
  - If you withdraw before the interview is transcribed and anonymized we won't use your interview for analysis or anonymous quotes in reports for the findings of the interview study

- Do you consent to taking part in the interview?

- Are you happy for me to record this?

- If no: let the participant know that more notes will be taken and the interview may last slightly longer.

Questions and prompts for all participants:

1. How did you find taking part in the SAFA study?
   Prompts:
   a. Why did you decide to take part?
   b. How did you feel about it overall?
   c. Can you tell me about anything you liked about the study?
   d. Can you tell me about anything you disliked about the study?

2. Where did you hear about the study?
   a. What did you think when you originally saw the invitation / advertisement?
   b. What did you expect when you first heard about the SAFA study?
   c. If you found out about the study on social media, what did you think of the signing-up process via social media?
   d. Can you think of any other ways we could have advertised the study to make more people aware of it?
   e. Are there any ways we could improve the recruitment process to encourage more people like you to take part?

3. What was your experience of the appointments that you had for the study, or the video or telephone appointments?
   Prompts:
   a. What suggestions do you have for improving the video or telephone appointments? (if relevant)
   b. Did you experience delays in getting your study medicine?

4. What was your experience of taking the study medicine?
   Prompts:
   a. How did you get on with the medicine?
   b. Did you have any side-effects? If so, what happened?
   c. Did you ever miss taking any tablets? If so, what happened? And how many times/how many tablets, approximately?
   d. How did you feel about the possibility of being given spironolactone or the placebo/dummy tablets? Did you guess which one you were taking?

5. How did you find the questionnaires?
   Prompts:
   a. Can you tell me about anything you liked about the questionnaires? Can you tell me about any difficulties you had completing the questionnaires?
   b. Can you remember how you found it answering the questions about your use of health services for your acne? Did you find it easy or difficult to remember at all?

c. Can you remember how you found it answering the questions about money you or your family had spent on acne? Did it cover all relevant costs? Did you find it easy recording costs for items like transport to appointments, etc?

For unblinded participants only:

6. Since stopping taking the tablets, have you wanted to start / continue spironolactone for your acne?
   Prompts:
   a. Have you asked a doctor to prescribe spironolactone?
   b. Did you experience any difficulties getting a prescription for spironolactone to treat your acne after you finished taking the study tablets?
   c. Did you ask your GP surgery or usual doctor to prescribe you spironolactone for your acne?

7. Since stopping taking the study tablets, have you used NHS services for your acne? This includes seeing a NHS doctor or nurse or dietician, going to hospital, or A&E.
   Prompts:
   a. Did you see a GP/practice nurse/dietician because of your acne?
   b. Have you visited a hospital as an outpatient because of your acne or side effects from treatment for your acne?
   c. Did you attend A&E because of your acne or side effects from treatments for your acne?
   d. Have you been admitted to hospital as an inpatient as a result of your acne or side effects from treatment for your acne?

8. During the Covid-19 pandemic, did you have difficulties accessing health services or getting medication for your acne?

Is there anything that we haven't discussed that you would like to add?

Would you like to see a copy of the report when we have finished the interview study?

Thank you for taking part in this interview.
