# Supplementary material for: Factors that influence women’s enrolment and ongoing participation in a partially decentralised randomised controlled dermatology trial: a qualitative interview study with participants in the SAFA (Spironolactone for Adult Female Acne) trial
Source: Trials. 2023 Oct 12;24:661. doi: 10.1186/s13063-023-07630-4 (PMC10568833; doi:10.1186/s13063-023-07630-4)
Supplement: Supplementary file 2 — Additional file 2. [file SAFA AM17 HRA approvalR2.pdf › SAFA AM17 HRA approvalR2.pdf]

Susanne Renz

**From:**	Wales.REC3@wales.nhs.uk <noreply@harp.org.uk>
**Sent:**	09 August 2021 10:19
**To:**	Miriam Santer; safa
**Subject:**	IRAS Project ID 246637. HRA and HCRW Approval for the Amendment
**Categories:**	Key SAFA emails

CAUTION: This e-mail originated outside the University of Southampton.

Dear Dr Santer,

**IRAS Project ID:**	246637
**Short Study Title:**	Spironolactone for Adult Female Acne
**Amendment No./Sponsor Ref:**	(ERGO45359.A20) - AM17
**Amendment Date:**	20 July 2021
**Amendment Type:**	Substantial CTIMP - for review

I am pleased to confirm **HRA and HCRW Approval** for the above referenced amendment.

You should implement this amendment at NHS organisations in England and Wales, in line with the guidance in the amendment tool.

User Feedback

The Health Research Authority is continually striving to provide a high quality service to all applicants and sponsors. You are invited to give your view of the service you have received and the application procedure. If you wish to make your views known please use the feedback form available on the HRA website: http://www.hra.nhs.uk/about-the-hra/governance/quality-assurance/.

Please contact amendments@hra.nhs.uk for any queries relating to the assessment of this amendment.

Kind regards

Miss Joanne Love - Wales
Approvals Administrator
Health Research Authority
Ground Floor | Skipton House | 80 London Road | London | SE1 6LH
E.amendments@hra.nhs.uk
W. www.hra.nhs.uk

Sign up to receive our newsletter HRA Latest.
